# P-979. Clinician Willingness to Consider Long-Acting Therapies and Culturally Supportive Strategies in Caring for Heavily Treatment-Experienced Black, Indigenous, or People of Color (BIPOC) with HIV

**DOI:** 10.1093/ofid/ofae631.1169

**Published:** 2025-01-29

**Authors:** Sumera Ackbarali, Sheldon D Fields, Mamta K Jain, Onyema Ogbuagu

**Affiliations:** PlatformQ Health Education, Lake Worth, Florida; The Pennsylvania State University, University Park, Pennsylvania; UT Southwestern Medical Center, Dallas, Texas; Yale School of Medicine, Cheshire, CT

## Abstract

**Background:**

Black, Indigenous, or People of Color (BIPOC) are overrepresented among heavily treatment-experienced (HTE) people with HIV (PWH). They face complex treatment challenges, exacerbated by socioeconomic disadvantages, and cultural insensitivities in healthcare settings that lead to suboptimal care and outcomes. To improve clinician competence in integrating long-acting agents into practice and providing culturally supportive care, an educational web-based initiative was designed in collaboration with the Association of Nurses in AIDS Care (ANAC).

**Figure:**
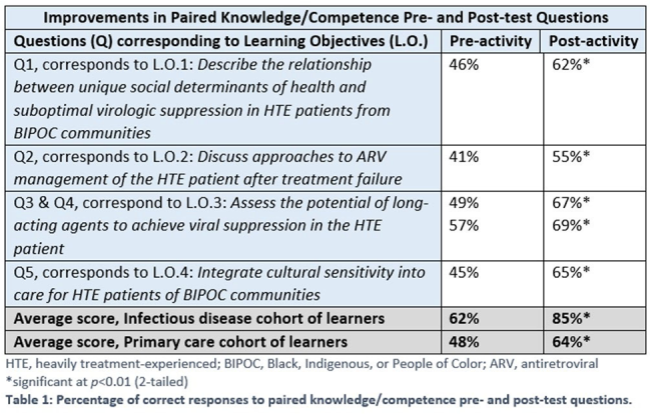

**Methods:**

A 60-minute CME activity, led by a multidisciplinary expert panel, was broadcast live on ClinicalSeriesLive and IDCareLive in August 2023 (on-demand until August 2024). Two vignettes of the treatment journey of HTE patients from BIPOC communities (Hispanic & African-American) were utilized to elevate awareness of unique challenges with care. Changes on knowledge/competence pre-/post-test questions and clinician perspectives and challenges (5-point Likert scale) were assessed.

**Results:**

As of April 30th, 2024, 1,728 clinicians participated in these activities (92% were physicians, advanced practitioners, and nurses). Prior to the activity, only 38% of learners had prescribed or administered a long-acting antiviral agent for HTE patients. Patients’ ability to access long-acting antiretroviral (ARV) therapy was the most frequently cited barrier by clinicians when considering this option (43%). Regarding culturally supportive care pre-activity, 12% of learners sought to understand stigma faced by patients within their communities and 11% spoke patients’ languages or referred them to translation services. Post-activity, learners demonstrated increased confidence in selecting a long-acting therapy (59%), discussing options with HTE patients (64%), and developing strategies to deliver more culturally supportive care (73%). Improvements in pre/post paired responses to CME questions are shown in Table 1.

**Conclusion:**

Web-based education on strategies to incorporate long-acting ARV therapies and culturally supportive care can improve clinician knowledge, uncover barriers, and stimulate practice change that improves care of HTE PWH from BIPOC communities.

**Disclosures:**

**Mamta K. Jain, MD, MPH**, Abbvie: Grant/Research Support|Gilead Sciences: Grant/Research Support|Laurent: Grant/Research Support **Onyema Ogbuagu, MD**, Gilead Sciences, Inc.: Advisor/Consultant|Gilead Sciences, Inc.: Honoraria|GSK/ViiV: Advisor/Consultant|GSK/ViiV: Honoraria|Janssen: Advisor/Consultant|Moderna: Advisor/Consultant|Moderna: Honoraria

